# Disease interception with interleukin-17 inhibition in high-risk psoriasis patients with subclinical joint inflammation—data from the prospective IVEPSA study

**DOI:** 10.1186/s13075-019-1957-0

**Published:** 2019-07-26

**Authors:** Eleni Kampylafka, David Simon, Isabelle d’Oliveira, Christina Linz, Veronika Lerchen, Matthias Englbrecht, Juergen Rech, Arnd Kleyer, Michael Sticherling, Georg Schett, Axel J. Hueber

**Affiliations:** 1 Department of Internal Medicine 3 – Rheumatology and Immunology, Friedrich-Alexander-University Erlangen-Nurnberg (FAU) and Universitaetsklinikum Erlangen, Ulmenweg 18, 91054 Erlangen, Germany; 2 Department of Dermatology, Friedrich-Alexander University Erlangen-Nurnberg (FAU) and Universitaetsklinikum Erlangen, Erlangen, Germany; 3 Sektion Rheumatologie, Sozialstiftung Bamberg, Bamberg, Germany

**Keywords:** Psoriasis, Psoriatic arthritis, Interleukin-17, Magnetic resonance imaging

## Abstract

**Background:**

A specific subset of psoriasis patients is characterized by subclinical inflammatory changes. These patients frequently present with arthralgia and have a higher risk to develop psoriatic arthritis (PsA). We hypothesized that IL-17A inhibition in this subset of patients can intercept the link between skin and joint disease and resolves pain and inflammatory changes.

**Methods:**

Psoriasis, but no PsA, patients were included in the open prospective exploratory Interception in very early PsA (IVEPSA) study. Patients had to have nail or scalp involvement or a high psoriasis area severity index (PASI) (> 6) as well as inflammatory or erosive changes in MRI or CT. Patients received treatment with the anti-interleukin (IL)-17A antibody secukinumab over 24 weeks. Clinical assessments of skin and joint disease were done at baseline and after 12 and 24 weeks, MRI and CT at baseline and after 24 weeks.

**Results:**

Twenty patients were included, 85% of them reporting arthralgia and 40% had tender joints at the examination. Eighty-three percent had at least one inflammatory lesion in the MRI, most of them synovitis/enthesitis. Skin disease (PASI: *p* < 0.002; BSA: *p* < 0.003) and arthralgia (VAS pain: *p* < 0.003) significantly improved after 24 weeks. Total PsAMRIS (*p* = 0.005) and synovitis subscore (*p* = 0.008) also significantly improved. Erosions and enthesiophytes did not progress, while bone mass in the distal radius significantly (*p* = 0.020) increased after 24 weeks.

**Conclusions:**

These data suggest that very early disease interception in PsA is possible leading to a comprehensive decline in skin symptoms, pain, and subclinical inflammation. IVEPSA therefore provides rationale for future early interventions with the concept to prevent the onset of PsA in high-risk individuals.

**Trial registration:**

Trial registry name PSARTROS; trial registry number: NCT02483234; June 26, 2015.

## Introduction

Psoriatic arthritis (PsA) is a chronic inflammatory disease affecting the joints and entheses, leading to tissue damage [1]. In the joints, the inflammatory process in PsA afflicts the synovium and the periosteal insertions of tendons and ligaments, leading to erosions and enthesiophytes, respectively [2]. The overwhelming knowledge of PsA comes from studies in established disease studying patients with several years of disease duration. Current knowledge on early disease however is still scarce. Nonetheless, a limited number of studies suggest that early PsA is different from established disease showing only moderate joint swelling but substantial pain and prevalence of enthesitis [3–6]. Furthermore, early treatment or even prevention of PsA may result in better outcomes for the patients [7, 8]. Understanding the biology of early disease in PsA is of utmost importance, as it will allow earlier interference with the disease process and better prevention of damage and disability [9]. This concept becomes even more important if considering that currently available intervention strategies for psoriatic disease have varying effects on the skin and the joints [10].

It seems that the onset of PsA is preceded by a prodromal phase (“transition phase”), in which the disease starts to spread from the skin to the joints [11]. This state, which is characterized by pain and joint tenderness—but no swelling—as well as the presence of distinct inflammatory joint lesions in the imaging analysis, is associated with a high risk to develop PsA [12, 13]. Based on these findings, this prodromal phase of the disease would allow developing strategies for early disease interception with the ultimate goal to prevent the onset of PsA. Before such strategies can be developed, however, it has to be clarified whether the symptoms and subclinical inflammatory joint lesions are sensitive to therapeutic intervention in principle.

The mechanisms of spreading of inflammatory skin disease to the joints are currently unknown. Nonetheless, some evidence suggests that interleukin (IL)-17A plays a role in this process: (i) Thus, overexpression of IL-17 in the skin does not only lead to psoriasis but also precipitate bone marrow activation and neutrophil recruitment [14]; (ii) IL-17 producing cells have been shown in the circulation of PsA patients and their number is associated to disease activity in the joints [15]; and (iii) IL-17 inhibition has shown to provide comprehensive effects on skin, entheseal, and joint manifestations of the disease [16, 17]. In addition to these observations, IL-17 has key effector functions in the joints such as osteoclast differentiation and bone erosions as well as most likely initiating altered stress responses in the joints leading to enthesiophyte formation [18, 19]. These structural effects of IL-17 have not only been substantiated in radiographic outcomes from the large randomized controlled clinical trials [20, 21] but also by smaller but more in-depth studies [22, 23] that showed an arrest of both catabolic and anabolic bone changes upon IL-17 inhibition.

Based on these data, we aimed to collect evidence for the principled possibility of early disease interception in the prodromal phase of PsA, by exposing psoriasis patients with active skin disease and subclinical joint inflammation in the MRI to anti-IL-17 antibody treatment with secukinumab for a total period of 24 weeks. We hypothesized that IL-17 inhibition resolves musculoskeletal pain and the early inflammatory changes in the joints of psoriasis patients.

## Patients and methods

### Study design and patients

The IVEPSA study (Interception in very early PsA) is a single-arm prospective exploratory open-label study to assess the effects of secukinumab treatment on the inflammatory and structural changes in the peripheral joints of psoriasis (and not PsA) patients. The protocol has been submitted as part of the PSARTROS study program (NCT02483234; June 2015) receiving ethics approval from the institutional review board (IRB) and the regulatory authorities. All patients provided written informed consent. Eligible patients had to have either moderate to severe psoriasis (PASI score > 6) or scalp or nail involvement. They also had to have inflammatory or erosive changes in MRI or erosive changes in the high-resolution peripheral quantitative computed tomography (HR-pQCT), but no clinical signs of arthritis at baseline. Hence, the included patients also did not fulfill the CASPAR criteria of PsA. Patients receiving treatment with systemic glucocorticoids or conventional or biological disease-modifying anti-rheumatic drugs (DMARDs) were not allowed into IVEPSA. Previous use of conventional DMARDs for the treatment of psoriasis was allowed, while previous use of biological DMARDs was not allowed. Also, other conditions such as recent live vaccines, history of tuberculosis, pregnancy, use of opioid analgesics, or drug abuse during the last 6 months or any uncontrolled medical condition were not allowed. If eligible, patients started to receive subcutaneous treatment with 300 mg secukinumab once weekly for the first months and once monthly thereafter for a total period of 24 weeks.

### Clinical assessments

Demographic parameters such as age, sex, and body mass index were recorded at baseline. The extent of skin disease was assessed by the psoriasis area severity index (PASI) and body surface area (BSA). All patients were asked for the presence/absence of arthralgia and joint tenderness as well as the severity of pain (visual analogue scale). In addition, patients’ and physicians’ ratings of global disease activity were recorded. Clinical examination was done for tender joint count (TJC) 78, swollen joint count 76, and SPARCC entheseal sites. In addition, the presence/absence of dactylitis and nail involvement was recorded. Per definition, patients were not allowed to have joint swelling, clinical signs of enthesitis, or presence of dactylitis at baseline. With respect to the impact of skin and musculoskeletal symptoms on patients’ quality of life, the dermatology quality of life index (DLQI) and the psoriatic arthritis impact of disease (PSAID) questionnaire, respectively, were used.

### Magnetic resonance imaging

MRI was used to assess inflammatory and structural changes in the joints. MRI scans of the dominant hand were performed at baseline and after 24 weeks of secukinumab treatment using a 1.5-T Magneton Avanto system (Siemens, Erlangen, Germany) as described before [22]. T1-weighted images with and without contrast agent as well as T2-weighted coronal fat saturated (TIRM) sequences were assessed for synovitis, periarticular inflammation, tenosynovitis, and bone erosions, bone proliferations, and osteitis. Images were evaluated by two independent assessors (IO, CL) blinded for the identity of the patients and the sequence of the images using standardized PsAMRIS-OMERACT scoring [24].

### High-resolution peripheral quantitative computed tomography

HR-pQCT was used to assess the structural changes in the joints (MCP and PIP) and volumetric bone mineral density (vBMD) and biomechanical parameters in the distal radius. HR-pQCT of the dominant hand was performed at baseline and after 24 weeks of secukinumab treatment using an XtremeCT I scanner (Scanco Medical). Scans were performed at the metacarpophalangeal (MCP) joints 2–4, the proximal interphalangeal (PIP) joints 2 and 3, and the distal radius. MCPs and PIPs were evaluated for erosions (numbers, volume) and enthesiophytes (grade 1: maximum height ≤ 4 mm; grade 2: maximum height > 4 mm; grade 3: diffuse osteoproliferation) as described previously [22]. Bone mass was assessed in the distal radius including the measurement of total, trabecular, and cortical vBMD (mg hydroxyapatite (HA) per cm^3^) as previously described [25]. For μFEA, finite element analysis software (FAIM, Version 8.0, Numerics88 solution, Calgary, Canada) was used. In order to generate micro-finite element models, the segmented trabecular network and cortex of the HR-pQCT images were used [26, 27]. Mesh size of the resulting models ranged from 1.5 to 3.5 million equally sized brick elements. Single linear isotropic tissue modeling was applied by assigning a tissue modulus of 6829 MPa and a Poisson’s ratio of 0.3 homogeneously to each element [28]. A linear uniaxial compression test was simulated. Nodes on the proximal bone surface were fixed in the *z* direction but unconstrained in *x* and *y* directions. Nodes on the distal bone surface were also free in *x* and *y* directions but exposed to a displacement equivalent to 1% strain along the *z*-axis [28]. Axial bone stiffness (kN/mm) as reaction force (RFz) divided by average displacement of the distal surface (Uz) and bone strength as estimated failure load (N) based on the Pistoia criterion was calculated [29].

### Statistical analysis

The hypothesis of the study was that secukinumab treatment leads to a significant improvement of (i) MRI synovitis and periarticular inflammation and (ii) pain and joint tenderness after 24 weeks. Wilcoxon signed-rank test was used for paired comparisons between baseline and week 24. Cross-sectional analyses were performed using the Mann-Whitney *U* test for differences and the Spearman correlation for relations. Due to the exploratory nature of the study, no sample size calculation was made. Statistical significance was set at *p* ≤ 0.05, data presented as median and quartiles. All analyses were performed in a two-tailed manner using IBM SPSS version 21.

## Results

### Baseline characteristics of the psoriasis patients

Twenty psoriasis patients were prospectively included in the study; 70% of them were males. Median age was 49.5 years (IQR 42.8, 59) and median disease duration 14 years (IQR 5, 20). Patients had moderate-to-high psoriasis activity at baseline according to PASI (median 6.9; IQR 3.5, 18.6) and BSA (median 10.9%; IQR 3.6%, 20.3%). Nail involvement was present in 55% and scalp involvement in 75% of the patients. Reported arthralgia was common at baseline and occurred in 85% (17/20) of patients. It was mostly based on inflammatory arthralgia (15/17); however, intensity was low with median VAS pain score of 18.5 (IQR 11.7, 49.5). The majority of patients with arthralgia (80%) reported an affection of the dominant hand, on which imaging was performed. Forty percent of patients had tender joints at examination (median TJC78 0, IQR 0, 3.8), but no patient had joint swelling. Patient demographics and clinical characteristics are summarized in Table 1. No patient had concomitant conventional DMARD or systemic steroid treatment during the study. Twenty-five percent of the patients had received previous treatment with methotrexate, and 20% were previously treated with dimethyl fumarate. One patient discontinued the treatment due to lack of efficacy at week 12, and another patient could not perform the follow-up MRI.

**Table 1:** Demographics and baseline clinical characteristics

Demographics	
Sex (male, %)	70%
Age (median, IQR)	49.5 (42.8, 59)
Disease duration (median, IQR)	14 (5, 20)
Clinical characteristics	
PASI (median, IQR)	6.8 (3.5, 18.6)
BSA% (median, IQR)	10.9 (3.6, 20.3)
Arthralgia (%)	85%
VAS_pain (median, IQR)	18.5 (11.75, 49.5)
VAS_patient global disease activity (median, IQR)	68 (45, 74)
VAS_physician (median, IQR)	35.5 (8.75, 62.75)
Tender joints (%)	40%
TJC78 (median, IQR; mean ± SD)	0 (0, 3.75); 2.65 ± 5.11
Nail involvement (%)	55%
Scalp involvement (%)	75%

Data are based on all 20 psoriasis patients

*IQR* interquartile range, *SD* standard deviation, *PASI* psoriasis area severity index, *BSA* body surface area, *VAS* visual analogue scale, *TJC* tender joint count

### Baseline imaging features of the psoriasis patients

**Fig. 1:**
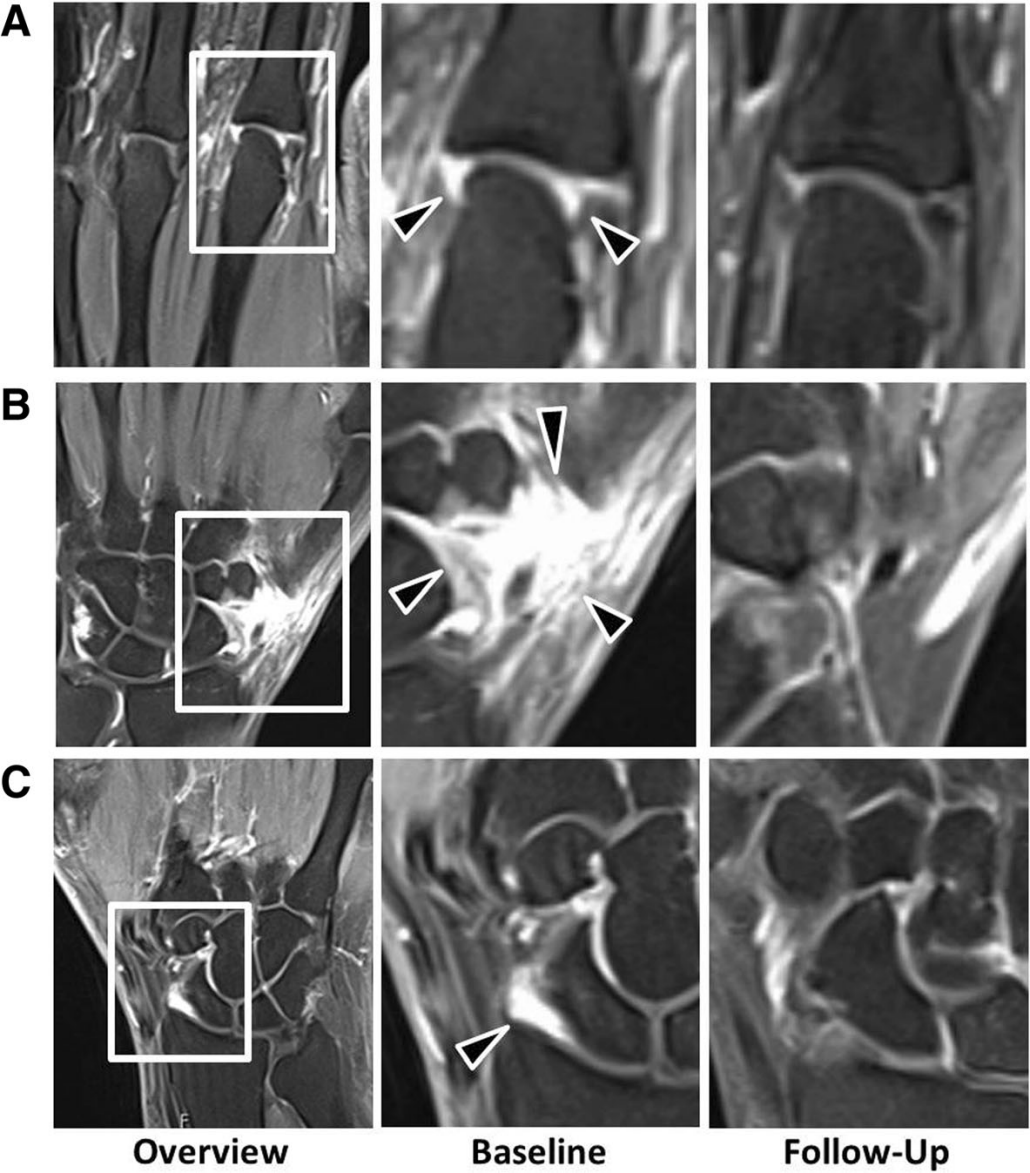
**a**–**c** Effects of secukinumab on signs of MRI inflammation in psoriasis patients at risk to develop PsA. T2-weighted coronal fat saturated (TIRM) sequences of the hands of psoriasis patients at risk to develop PsA. Three examples, one from metacarpophalangeal joints and two from the wrist joint, are shown. Left column: overview at baseline; middle column: close-up of the inflammatory lesion at baseline; right column: follow-up of the same region after 24 weeks of secukinumab treatment. Black arrows mark the lesion; white frames mark the area depicted in the close-up

Baseline MRI investigation revealed at least one inflammatory lesion in 83.3% of patients (for examples, see Fig. 1). Synovitis was the most prevalent (66.7%), followed by tendinitis (55.6%), osteitis (27.8%), and periarticular inflammation (16.7%). Median total PsAMRIS score was 2.5 (IQR 0, 6). Erosions were present in 72.2% and 56.3% in the MRI and HR-pQCT, respectively, and enthesiophytes were found in 33.3% and 37.5%, respectively. More specifically, all enthesiophytes demonstrated in HR-pQCT were graded either as mild (grade 1, 23.5%) or as moderate (grade 2, 17.6%). No cases of severe diffuse osteoproliferation were recorded. All baseline imaging data are presented in Table 2.

**Table 2:** Baseline imaging characteristics

MRI	
Synovitis (%)	66.7%
PSAMRIS synovitis (median, IQR)	1 (0, 2)
PSAMRIS synovitis (mean ± SD)	2.17 ± 4.6
Osteitis (%)	27.8%
PSAMRIS osteitis (median, IQR)	0 (0, 0)
PSAMRIS osteitis (mean ± SD)	0.44 ± 1.89
Erosion (%)	72.2%
PSAMRIS erosion (median, IQR)	1 (0, 4)
PSAMRIS erosion (mean ± SD)	2.56 ± 3.7
Proliferation (%)	33.3%
PSAMRIS proliferation (median, IQR)	0 (0, 1)
PSAMRIS proliferation (mean ± SD)	0.44 ± 0.71
Periarticular (%)	16.7%
PSAMRIS periarticular (median, IQR)	0 (0, 0)
PSAMRIS periarticular (mean ± SD)	0.28 ± 0.67
Tenosynovitis (%)	55.6%
PSAMRIS tenosynovitis (median, IQR)	0 (0, 1)
PSAMRIS tenosynovitis (mean ± SD)	0.61 ± 1.15
Total PSAMRIS (median, IQR)	4 (0.75, 7.25)
Total PSAMRIS (mean ± SD)	6.5 ± 8.85
HR-pQCT	
Erosions (%)	58.8%
Erosion no. (median, IQR)	1 (0, 1.75)
Enthesiophytes (%)	41.2%
Grade 1	23.5%
Grade 2	17.6%
Grade 3	0%

Data are based on 18 psoriasis patients who completed the study

*PSAMRIS* psoriatic arthritis magnetic resonance imaging scoring system, *MRI* magnetic resonance imaging, *HR-pQCT* high-resolution peripheral quantitative computed tomography, *IQR* interquartile range, *SD* standard deviation

### Effects of secukinumab treatment on psoriatic skin disease and musculoskeletal symptoms

**Fig. 2:**
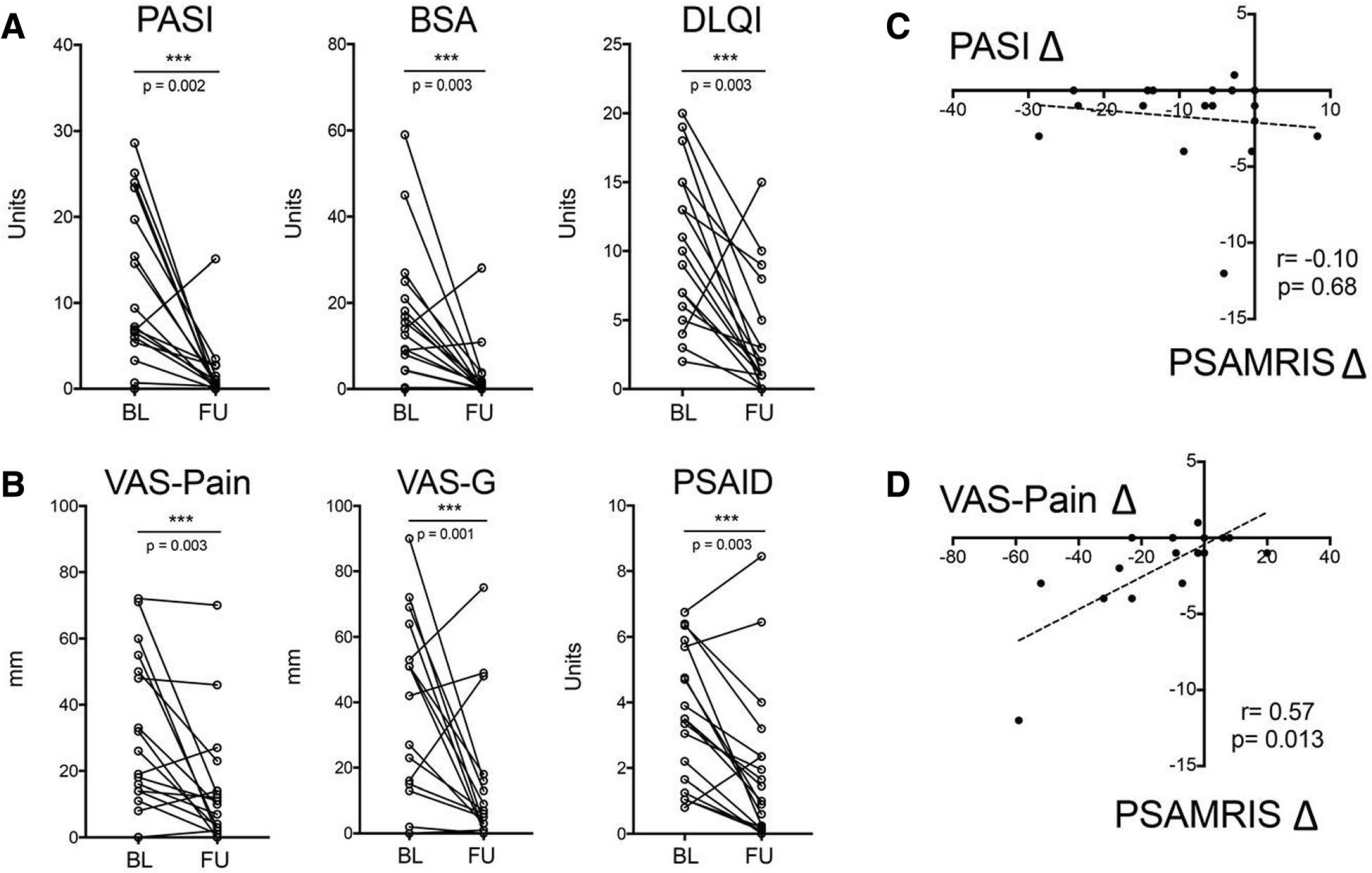
Effects of secukinumab on skin and musculoskeletal manifestations of psoriasis patients at risk to develop PsA. **a** Baseline and 24-week psoriasis area severity index (PASI), body surface area (BSA), and dermatology quality of life index (DLQI) in psoriasis patients at risk to develop PsA. **b** Baseline and 24-week visual analogue scale (VAS) for pain, VAS for global musculoskeletal disease activity, and psoriatic arthritis impact of disease (PSAID) in psoriasis patients at risk to develop PsA. **c** Correlation between total psoriatic arthritis magnetic resonance imaging score (PsAMRIS) and PASI score. **d** Correlation between total PsAMRIS and VAS for pain

Psoriatic skin disease (total PASI and BSA) significantly improved after 24 weeks of secukinumab treatment (Table 3; Fig. 2a): PASI significantly (*p* = 0.002) declined from a median score of 6.9 at baseline to 0.4 after 24 weeks. BSA also significantly (*p* = 0.003) decreased from a median of 10.9% at baseline to 0.2% at follow-up. In accordance, skin disease-related quality of life of the patients measured by improved DLQI significantly (*p* = 0.0009) improved. Extent of arthralgia significantly (*p* = 0.003) declined after secukinumab treatment from a median of 18.5 mm at baseline to a median of 7 mm at follow-up (Table 3; Fig. 2b). In accordance, we observed a significant improvement of patients global (*p* = 0.003) as well as a significantly (*p* = 0.0014) lower impact of musculoskeletal symptoms on patients’ quality of life as measured by the PSAID. In patients with MRI inflammation at baseline, also the number of painful joints at baseline (1, IQR 0, 4) significantly improved at week 24 (0, IQR 0, 2; *p* = 0.038) (Table 3). No patient developed PsA during the observation period.

**Table 3:** Changes in signs and symptoms of psoriasis between baseline and week 24

	Baseline	Week 24	*p* value
Characteristic (*n* = 18)			
PASI (median, IQR)	6.9 (3.5, 18.6)	0.4 (0, 1.5)	0.002**
BSA% (median, IQR)	10.9 (3.6, 20.3)	0.2 (0, 1.8)	0.003**
DLQI (median, IQR)	10 (5, 15)	1.5 (0.75, 5.75)	0.003**
VAS pain (median, IQR)	18.5 (11.75, 49.5)	7 (1, 14)	0.003**
VAS global (median, IQR)	68 (45, 74)	8 (2.5, 26)	< 0.001**
VAS physician (median, IQR)	35.5 (8.75, 62.75)	7 (1, 15)	0.011*
TJC 78 (median, IQR) (mean ± SD)^†^	0 (0, 3.75); 2.65 ± 5.11	0 (0, 2); 0.63 ± 1.17	0.102
PsAID (median, IQR)	3.5 (1.65, 1.7)	1.35 (0.2, 2.35)	0.003**
Characteristic (*n* = 15)			
PASI (median, IQR)	6.9 (3.3, 15.4)	0.3 (0, 1.1)	0.007**
BSA% (median, IQR)	9.2 (4.3, 18.1)	0.05 (0, 1)	0.013*
DLQI (median, IQR)	12 (6.75, 15.75)	2 (0.75, 8.25)	0.013*
VAS pain (median, IQR)	26 (14, 50)	4 (1, 23)	0.004**
VAS global (median, IQR)	70 (61, 75)	12 (1, 38)	0.001**
VAS physician (median, IQR)	37 (7, 50)	4 (1, 22)	0.036*
TJC 78 (median, IQR) (mean ± SD)^†^	1 (0, 4); 3.53 ± 5.67	0 (0, 2); 0.53 ± 0.92	0.038*
PsAID (median, IQR)	3.7 (2.84, 6.01)	1.45 (0.25, 3.2)	0.005**

Data on the upper half are based on 18 psoriasis patients with complete baseline and 24-week data. Data on the lower half are based on patients with active joint inflammation in MRI at baseline (*n* = 15)

*TJC* tender joint count, *VAS* visual analogue scale, *PASI* psoriasis area and severity index, *BSA%* percent body surface area, *IQR* interquartile range, *DLQI* dermatology life quality index, *PsAID* psoriatic arthritis impact of disease

^†^Additionally reported as mean and standard deviation (mean ± SD) because median was equal to zero

Wilcoxon signed-rank test. *p ≤ 0.05, **p ≤ 0.01

### Effects of secukinumab treatment on subclinical inflammation and local bone changes

Sequential assessment of joint inflammation by MRI showed a significant decrease in global PsAMRIS (*p* = 0.005) and PsAMRIS synovitis score (*p* = 0.008) after 24 weeks of secukinumab treatment (Table 4). Furthermore, periarticular inflammation completely disappeared. Importantly, improvement in total PsAMRIS score significantly correlated with the improvement in arthralgia (VAS pain score difference, *p* = 0.009) but not with skin disease (Fig. 2c, d).

**Table 4:** Changes of the inflammatory and structural parameters between baseline and week 24

Characteristic	Baseline	Week 24	*p* value
MRI			
PSAMRIS synovitis (median, IQR) (mean ± SD)^†^	1 (0, 2); 2.17 ± 4.61	1 (0, 2); 1.06 ± 1.96	0.008**
PSAMRIS osteitis (median, IQR) (mean ± SD)^†^	0 (0, 0); 0.44 ± 1.89	0 (0, 0); 0.33 ± 1.41	0.317
PSAMRIS periarticular (median, IQR) (mean ± SD)^†^	0 (0, 0); 0.28 ± 0.67	0 (0, 0); 0 ± 0	0.102
PSAMRIS tenossynovitis (median, IQR) (mean ± SD)^†^	0 (0, 1); 0.61 ± 1.15	0 (0, 0); 0.33 ± 0.84	0.096
PSAMRIS erosions (median, IQR)	2 (0, 4)	2 (0, 4)	1.000
PSAMRIS proliferation (median, IQR) (mean ± SD)^†^	0 (0, 1); 0.44 ± 0.71	0 (0, 1); 0.44 ± 0.86	1.000
Total PSAMRIS (median, IQR)	5 (1, 8)	3 (0, 6)	0.005**
HR-pQCT			
Erosion number (median, IQR)	1 (0, 1.75)	1 (0, 1.75)	0.317
Proliferation grade (median, IQR) (mean ± SD)^†^	0 (0, 1); 0.55 ± 0.76	0 (0, 2); 0.68 ± 0.89	0.180
Dtot, HA/cm^3^ (median, IQR)	303.1 (264.8, 320.8)	308.9 (265.8, 324.9)	0.020*
Dtrab, HA/cm^3^ (median, IQR)	165.2 (133.5, 205.7)	165.7 (136.9, 206.9)	0.048*
Dcomp, HA/cm^3^ (median, IQR)	840.5 (778.8, 848.3)	831.1 (796.5, 854.8)	0.184
Bone stiffness, kN/mm (median, IQR)	48.38 (38.58, 60.31)	49.40 (38.14, 59.82)	0.501
Failure load, N (median, IQR)	2312.2 (1862.6, 2817.0)	2390.0 (1798.6, 2847.2)	0.352

Data are based on 18 psoriasis patients with complete baseline and 24-week data

*PSAMRIS* psoriatic arthritis magnetic resonance imaging scoring system, *MRI* magnetic resonance imaging, *HR-pQCT* high-resolution peripheral quantitative computed tomography, *IQR* interquartile range, *Dtot* average bone density, *HA* hydroxyapatite, *Dtrab* trabecular bone density, *Dcomp* cortical bone density, *N* Newton

^†^Additionally reported as mean and standard deviation (mean ± SD) because median was equal to zero

Wilcoxon signed-rank test. *p ≤ 0.05, **p ≤ 0.01

We also examined whether secukinumab therapy arrests the progression of structural damage in psoriasis patients. Bone erosions were assessed at baseline and after 24 weeks of secukinumab treatment using MRI and HR-pQCT. In MRI and in HR-pQCT, PsAMRIS erosion score and numbers respectively remained stable over the 24 weeks of treatment with no signs of progression (Table 4). Interestingly, patients with erosions presented with higher level of arthralgia at baseline and after secukinumab treatment (week 24) (*p* = 0.044 and 0.025, respectively). We also checked the progression of enthesiophytes, which are a hallmark of structural damage in psoriasis. In the MRI, where osteoproliferation is also scored, no progression was found. In the HR-pQCT, which is more sensitive for enthesiophytes, no progression of proliferation numbers or grade was detected during the treatment with IL-17 inhibitor. Overall, no progression of catabolic and anabolic bone changes was found during the treatment in this prodromal phase of PsA.

### Effects of IL-17 blockade on bone structure and biomechanical properties

We finally investigated bone volumetric density (vDMD) and biomechanical properties at the distal radius. Small but significant increase of vBMD was observed from baseline (303.1 HA/cm^3^) to follow-up (308.9 HA/cm^3^; *p* = 0.020) (Table 4). Also, trabecular vBMD significantly improved from baseline (165.2 HA/cm^3^) to follow-up (165.7 HA/cm^3^; *p* = 0.048). Cortical vBMD remained stable, and also, biomechanical bone parameters such as failure load and stiffness resembling biomechanical properties of the bone remained stable (Table 4).

## Discussion

Transition from psoriatic skin disease to PsA is an important step as PsA is associated with substantially higher disease burden due to pain and functional impairment. Furthermore, structural changes accrue in PsA, representing an irreversible damage to tissue architecture in the joints and in the entheses. It is well known that psoriasis typically precedes the development of PsA, allowing defining a population at-risk for developing PsA. However, if looking at an unselected group of psoriasis patients, the progression rate to PsA is rather limited, necessitating the definition of specific subpopulations of psoriasis patients that show a high risk to develop joint disease [30]. Such populations may profit best from the early systemic treatment of the disease [10].

IVEPSA shows the principled feasibility to identify a subset of psoriasis patients, who show symptoms as well as subclinical signs of inflammation suggestive for the prodromal phase of PsA, and the possibility to treat such patients with systemic cytokine neutralization leading to regression of inflammatory lesions and improvement of joint pain. Psoriasis patients in this study had to have previously defined risk factors such as nail or scalp involvement or moderate to severe psoriasis [31]. However, the mere fulfillment of such risk factors may not be enough to sufficiently pin down the population with high risk for PsA. Therefore, we have built on previous data that showed that subclinical inflammatory lesions in the joints of psoriasis patients massively enhance the risk for the development of PsA, resembling a population that closely resembles the prodromal phase of PsA [7]. This subgroup of psoriasis patients is also characterized by a high prevalence of joint pain, showing parallels to observations made in RA, where pain and subclinical inflammatory lesions precede the onset of joint disease. Notably, none of our patients had signs of PsA such as joint swelling, clinical enthesitis, or dactylitis.

Inhibition of IL-1A by secukinumab not only significantly improved psoriatic skin lesions but also reduced pain and subclinical inflammatory lesions in psoriasis patients in the prodromal phase of PsA. This effect was accompanied by an improvement of quality of life with respect to skin and musculoskeletal manifestations of the disease. This observation suggests that joint pain as well as the development of the articular MR lesions depends on IL-17 activity. We think that this is an important finding as it indicates that symptoms and MR lesions in this prodromal phase of the disease are not unspecific in their nature but related to the disease process. This notion is in line with the observation of a significant relation between pain responses and the reduction of MR lesions.

Our data give support to the concept that selective interventions in a defined subset of psoriasis patients may be a feasible approach to prevent the onset of PsA. The concept to prevent PsA by interfering early in the process of psoriatic disease is appealing but challenging as the success of such studies depends on the appropriate intervention and the right selection of patients. An unselected approach in all-comers with psoriasis seems hardly feasible based on the large number of patients to be included and the long duration of such study. Therefore, rigorous selection of psoriasis patients that are in transition to PsA seems necessary, and this exploratory study provides the basis for such approach [10]. The limitation of this study is the small number of patients and the observational character without inclusion of placebo control. Therefore, results have to be interpreted with caution. Notably, this study has not been designed to answer the question if PsA can be prevented but rather tested the feasibility to identify a population of imminent PsA, treat such population with cytokine inhibitors, and show a biological effect on subjective and objective signs of inflammation. Based on these findings, larger randomized controlled studies can be designed.

Early intervention is yet an under-recognized clinical unmet need in PsA. Thus, virtually, all interventional studies to date have focused on established disease, in which substantial joint damage has already accrued. IVEPSA data show that even in this very early population, some catabolic and anabolic bone damage can be observed, suggesting the need for early intervention. The observation of a higher prevalence of erosions than enthesiophytes in this population was at first sight surprising, as previous studies revealed enthesiophytes as first musculoskeletal lesions in psoriasis [23, 32, 33], however, but may be owed to the fact that in this case a population with subclinical inflammatory lesions in the joints has been selectively studied. Furthermore, the data also show that inhibition of IL-17 arrests the progression of catabolic and anabolic changes in the joints of these patients. This finding is in accordance with the one obtained in established disease using high-end imaging, but the difference is that in the IVEPSA population, bone damage is halted at a much less progressed stage. Although the study design does not allow to judge whether structural progression would have occurred in the absence of treatment, previous data showed that HR-pQCT allows detecting structural progression within the same time frame in PsA patients treated with methotrexate or TNF inhibitors [34].

## Conclusions

### Feasibility of disease interception in very early psoriatic arthritis

Taken together, the data obtained in the IVEPSA study suggest that early therapeutic intervention during the transition phase from psoriasis to PsA is feasible and associated with improvement in the clinical and imaging manifestations of emerging PsA. Thereby, this study provides the rationale for early disease interception with the vision to prevent PsA.

## Data Availability

The datasets used and/or analyzed during the current study are available from the corresponding author on reasonable request.
